# Charakteristika und Wirksamkeit von Gesundheitslotsen in Projekten des Innovationsfonds: ein Scoping-Review

**DOI:** 10.1007/s00103-025-04177-4

**Published:** 2025-12-19

**Authors:** Lorenz Harst, Marie Coors, Helene Hense, Jamina Nagl, Tina Haase, Leonie Sundmacher, Jochen Schmitt

**Affiliations:** 1 Medizinische Fakultät und Universitätsklinikum Carl Gustav Carus, Zentrum für Evidenzbasierte Gesundheitsversorgung (ZEGV), Technische Universität Dresden, Fetscherstr. 74, 01307 Dresden, Deutschland; 2 School of Medicine and Health, Fachgebiet für Gesundheitsökonomie, Technische Universität München, München, Deutschland; 3 Munich Center for Health Economics and Policy (M-CHEP), München, Deutschland

**Keywords:** Koordinierte Versorgung, Case-Management, Patientenpfade, Sektorenübergreifende Versorgung, Effektivität, Coordinated care, Case management, Effectiveness, Patient pathways, Cross-sectional care

## Abstract

**Einleitung:**

Personen mit komplexen Versorgungsbedarfen, zum Beispiel mit chronischen und Mehrfacherkrankungen, sind auf eine kontinuierliche und sektorenübergreifende Behandlung angewiesen. Ein komplexer werdendes Gesundheitssystem mit einer strikten Trennung zwischen ambulanter und stationärer Versorgung erschwert diese Behandlungskontinuität und führt zu Über‑, Unter- und Fehlversorgung. Gesundheitslotsen sollen dieses Problem angehen, indem sie Betroffene gezielt durch das Gesundheitssystem navigieren.

**Methoden:**

Das vorliegende Scoping-Review gibt einen Überblick über die Zielgruppen, Aufgaben und Qualifikationen der Lotsen, Rechtsgrundlage sowie Wirksamkeit der seit 2016 durch den Innovationsfonds (Neue Versorgungsformen) geförderten Lotsenprojekte. Für das Scoping-Review wurden alle Lotsenprojekte in der Datenbank des Innovationsfonds, für die bis zum 01.04.2025 ein Abschluss- und Evaluationsbericht vorlag, analysiert.

**Ergebnisse:**

Die 31 eingeschlossenen Lotsenprojekte adressierten ein breites Spektrum an Zielgruppen. Am häufigsten wurden Personen in belastenden Lebenssituationen (*n* = 10), mit Herz-Kreislauf-Erkrankungen (*n* = 5), mit Erkrankungen der Psyche/Nerven (*n* = 5) und Stoffwechselerkrankungen (*n* = 3) adressiert. Die Projekte untersuchten 153 verschiedene Endpunkte. Am häufigsten zeigte sich kein Effekt. Signifikant positive Effekte wurden am häufigsten für Patient-reported Outcome Measures (PROMs) (*n* = 21), Prozessindikatoren (*n* = 19) und Patient-reported Experience Measures (PREM)s (*n* = 10) erzielt, negative für gesundheitsökonomische Outcomes im Sinne einer Kostensteigerung (*n* = 5).

**Diskussion:**

Eine Vereinheitlichung des Reportings zu Lotsenprojekten, die detaillierte Beschreibung der Lotsentätigkeiten sowie eine verstärkte Nutzung von Wirkmodellen könnten helfen, die Wirksamkeit von Lotsen in Zukunft besser zu verstehen.

**Zusatzmaterial online:**

Zusätzliche Informationen sind in der Online-Version dieses Artikels (10.1007/s00103-025-04177-4) enthalten

## Abstract (en)

**Introduction:**

Individuals with complex healthcare needs, such as those with chronic and multiple conditions, require continuous, cross-sectoral care. However, an increasingly complex healthcare system, characterized by a strict separation between outpatient and inpatient care, hampers continuity of care and contributes to overuse, underuse, and misuse of services. Healthcare navigators (“Gesundheitslotsen”) aim to address these challenges by supporting affected individuals in navigating the healthcare system.

**Methods:**

This scoping review provides an overview of the target populations, roles, and qualifications of navigators as well as the legal framework and effectiveness of navigators studied in projects funded by the German Innovation Fund (New Forms of Care) since 2016. All projects concerning navigators listed in the Innovation Fund database with a project report available as of 1 April 2025 were included in the analysis.

**Results:**

A total of 31 navigator projects were identified, addressing a wide range of target groups. The most frequently targeted populations included individuals in challenging life situations (*n* = 10) and with cardiovascular diseases (*n* = 5), mental or neurological disorders (*n* = 5), and metabolic conditions (*n* = 3). Across all projects, 153 distinct endpoints were evaluated. In most cases, no effect was observed. Statistically significant positive effects were most frequently reported for patient-reported outcome measures (PROMs) (*n* = 21), process indicators (*n* = 19), and patient-reported experience measures (PREMs) (*n* = 10). Negative effects were primarily observed in health economic outcomes, particularly in terms of increased costs (*n* = 5).

**Discussion:**

Standardizing reporting on pilot projects, concisely describing activities of patient navigators, and making greater use of impact models could help to improve understanding of the effectiveness of patient navigators in the future.

## Einleitung

Demografischer Wandel, Fachkräftemangel, Budgetdefizite, die Digitalisierung analoger Prozesse und das Nebeneinander von Unter‑, Über- und Fehlversorgung – die Liste der Anforderungen an ein modernes, resilientes deutsches Gesundheitssystem wächst stetig [1].

Die Zahl der vertragsärztlichen Behandlungsfälle in Deutschland wurde im Jahr 2022 auf 578 Mio. geschätzt. Das sind mehr als 8 Arztkontakte pro Einwohner, Tendenz steigend [2, 3]. Damit liegt Deutschland im internationalen Ranking der OECD auf Platz 4. Zusätzlich gab es in knapp 1900 Krankenhäusern rund 16,8 Mio. behandelte Fälle [4–6] – Platz 2 im internationalen Vergleich der OECD [5]. Darüber hinaus stellt die historisch gewachsene Trennung von ambulantem und stationärem Sektor einen Designfehler im deutschen Gesundheitswesen dar – insbesondere im Hinblick auf doppelte Diagnosen und Informationsverluste bei der Arzneimittelgabe, aber auch bzgl. der doppelten Facharztschiene bei gleichzeitigem Fachkräftemangel. Zeitgleich bestehen trotz der hohen Anzahl an Arztkontakten und stationären Fallzahlen sowie hoher Versorgungsdichte selbst in städtischen Regionen Zugangsprobleme, insbesondere für sozial benachteiligte Personengruppen [7, 8].

Die nur langsam voranschreitende Einführung der elektronischen Patientenakte (ePA) und die freie Arztwahl bringen einen gesteigerten inter- und intrasektoralen Koordinationsbedarf mit sich, welcher wiederum zu Doppeldiagnostik, ausbleibender Nachversorgung, Polymedikation und weiteren Schnittstellenproblemen führen kann [9]. Patienten mit geringer navigationaler Gesundheitskompetenz, aber auch besonders chronisch und schwer kranke Patienten sind auf eine kontinuierliche und sektorenübergreifende Behandlung angewiesen, die nur durch eine koordinierte Zusammenarbeit der involvierten Leistungserbringenden erreicht werden kann [10]. An dieser Stelle sollen Lotsen die kontinuierliche, patientenindividuelle und qualitativ hochwertige Begleitung von Patienten durch das deutsche Gesundheitssystem sicherstellen. Es handelt sich hierbei um ein Tätigkeitsfeld, das bereits in verschiedenen Projekten erprobt wurde und unterstützend zu den medizinischen Leistungen der Ärzte koordinierende, organisatorische und betreuende Aufgaben übernehmen soll. Personal, das im Gesundheitswesen diese Aufgaben übernimmt, empfahl der Sachverständigenrat Gesundheit & Pflege bereits 2007 [11]. Bisher hat sich keine einheitliche Begrifflichkeit durchgesetzt, weswegen in der breiten Fachöffentlichkeit die Begriffe Patientenlotse, Case oder Care Manager, Gesundheitslotse und Kümmerer Verwendung finden [12]. Der Begriff „Lotse“ wird im vorliegenden Beitrag als übergreifende Sammelbezeichnung genutzt.

Bundesweit existieren bereits über 55 Modellprojekte, die auf Grundlage des Case-Management-Regelkreises den Einfluss von Lotsen sowohl auf das Versorgungsgeschehen als auch auf ökonomische und klinische Outcomes untersuchen [13, 14]. Auffällig ist die große Bandbreite der adressierten Krankheitsbilder, denen jedoch allen ein komplexer, multidisziplinärer Versorgungsbedarf zugrunde liegt [12]. Im Zuge der Projektausschreibungen zu Neuen Versorgungsformen hat auch der Innovationsausschuss des gemeinsamen Bundesausschusses (G-BA) bereits mehrere Projekte aus dem Innovationsfonds gefördert, in denen der Einsatz von Lotsen in verschiedenen Settings erprobt wurde. Die vorliegende Arbeit bietet einen Überblick über diese Projekte und soll folgende Forschungsfragen beantworten:

1. Welche Patienten(gruppen) und/oder Betreuungspersonen wurden von Lotsen in den bisher abgeschlossenen Innovationsfondsprojekten adressiert?
2. Welche Aufgaben übernehmen Lotsen in den untersuchten Projekten?
3. Welche Qualifikationen haben die Lotsen in den untersuchten Projekten?
4. Anhand welcher Rechtsgrundlage erfolgte der Einsatz der Lotsen in den untersuchten Projekten?
5. Wie effektiv und effizient sind Lotsen in Bezug auf klinische, patientenberichtete, gesundheitsökonomische und prozessorale Outcomes?

## Methoden

Das vorliegende Scoping-Review beruht ausschließlich auf Ergebnis- und Evaluationsberichten der aus dem Innovationsfonds geförderten Projekte der Förderlinie Neue Versorgungsformen seit deren Einführung. Es ist der erste Teil des geplanten Systematic Reviews zum Einsatz von Lotsen („patient navigators“) in Deutschland (Registrierung und Details siehe [15]).

Entsprechend wurden sämtliche Projekte in die Betrachtung eingeschlossen, die Lotsen als Personen verstehen, die Patienten mit komplexen Gesundheits- und Versorgungsbedarfen und -bedürfnissen, z. B. aufgrund chronischer oder mehrfacher Erkrankungen, individuell beraten und unterstützen (in Anlehnung an [12–16]). Lotsen müssen nicht zwingend Ärzte oder Pflegekräfte sein, können diesen Berufsgruppen aber angehören. Projekte, bei denen die Aufgabe der Lotsen von Laien, etwa Peers der betroffenen Patienten, übernommen werden, wurden nicht berücksichtigt (z. B. PROMISE). Ebenso wurden Projekte ausgeschlossen, bei denen den Patienten anstelle einer unterstützenden Person lediglich Informationsmaterial zur Verfügung gestellt wurde oder die Koordination der Versorgung allein durch eine elektronische Patientenakte (ePA) oder ein digitales Angebot erleichtert werden sollte.

Es wurden keine Einschränkungen bzgl. des Krankheitsbilds der untersuchten Patienten vorgenommen, solange ein komplexer Versorgungsbedarf vorlag, der eine Koordination zwischen Leistungserbringenden und/oder anderen Gesundheitsleistungen erforderte. Lotsenprojekte oder deren Komponenten, die lediglich der Navigation von Personen durch Angebote der Primär- und Sekundärprävention dienten, wurden nicht berücksichtigt.

Ausschließlich Projektergebnisse mit Bezug zur Wirksamkeit des Einsatzes von Lotsen, gemessen entweder anhand von klinischen, patientenberichteten, prozessoralen oder gesundheitsökonomischen Outcomes, wurden für das vorliegende Review berücksichtigt. Es wurden keine Ergebnisse zur formativen (Implementierungs- oder Prozess‑)Evaluation betrachtet.

Weiterhin wurden nur Projekte in die Analyse eingeschlossen, für die zum 01.04.2025 ein finaler Abschluss- und Evaluationsbericht ebenso wie ein begründeter Beschluss des Innovationsausschusses bzgl. einer Empfehlung zur Implementierung der Intervention in die Regelversorgung vorlag. Die Projektdatenbank des Innovationsfonds (Förderlinie Neue Versorgungsformen) wurde vollständig anhand der oben gelisteten Kriterien von 2 Personen (HeHe und JN) unabhängig voneinander gesichtet. Wo Uneinigkeit bzgl. des Einschlusses eines Projekts bestand, wurde eine dritte Person (LH) hinzugezogen. Die Projektcharakteristika und -ergebnisse wurden anschließend anhand eines vorab pilotierten Datenextraktionsschemas von insgesamt 6 Personen (MC, JN, HeHe, LSS, JH, LH) extrahiert, wobei jede Extraktion stichpunktartig von einer zweiten Person geprüft wurde.

Für den vorliegenden Artikel wurden die untersuchten Populationen, Aufgaben der Lotsen und erhobenen Outcomes jeweils kategorisiert. Grundlage der Kategorisierung der Populationen waren die ICD-10-Codes, sofern konkrete Krankheitsbilder untersucht wurden. Zusätzlich wurden alle Projekte den Kategorien zugeordnet, die der Bundesverband Managed Care (BMC) seiner Lotsenlandkarte zugrunde gelegt hat [13]. Diese lauten: Geriatrie, Herz und Kreislauf, Onkologie, Psyche und Nerven, Multimorbidität, Versorgung in belastenden Lebenssituationen, Muskeln und Skelett, Stoffwechsel, seltene Erkrankungen und regionaler Versorgungsbedarf. Für nicht zuzuordnende Projekte wurde eine Kategorie „Sonstiges“ geschaffen. Die Aufgaben der Lotsen wurden in Anlehnung an die Struktur eines internationalen Scoping-Reviews zu „patient navigators“ kategorisiert [16].

Für Outcomes wurde eine Kategorisierung im Sinne der Forschungsfragen nach prozessoralen, patientenberichteten (PROM und PREM), gesundheitsökonomischen und klinischen Outcomes vorgenommen. In einzelnen Fällen wurden Outcomes als die patientenbezogene Versorgungssituation betreffend kategorisiert, da diese Informationen relevant waren, um patientenberichtete, gesundheitsökonomische und klinische Outcomes in Kontext zu setzen. Prozessorale Outcomes informieren zudem gesundheitsökonomische Ergebnisse einzelner Projekte, da sie etwa die Inanspruchnahme von Gesundheitsleistungen betreffen. Bei der Ergebnisdarstellung wurde zwischen primären und sekundären Outcomes der Studien unterschieden.

Zur systematischen Einordnung der Ergebnisse wurden die Interventionseffekte nach Effektrichtung und -stärke klassifiziert: signifikant positiv, signifikant negativ, positive Tendenz, negative Tendenz, unklare Wirksamkeit und kein Effekt. Die Richtung der Effekte wurde dabei im Sinne einer positiven Wirkung des Lotsenmodells interpretiert. Eine positive bzw. negative Tendenz wird berichtet, sofern diese von den Evaluatoren als solche benannt wurde. Kein Effekt liegt vor, wenn laut der Ergebnisberichte zwischen Interventions- und Kontrollgruppe kein Unterschied bestand. Unklare Wirksamkeit kam studienbedingt etwa durch den Ausfall einer Kontrollgruppe oder zu geringe Teilnehmendenzahlen ab einem bestimmten Follow-up-Zeitpunkt zustande. Bei der Zusammenfassung der Studienergebnisse ist zu beachten, dass sich Effekte zwischen den Follow-up-Zeitpunkten in ihrer Richtung unterscheiden können.

## Ergebnisse

Insgesamt wurden 99 Projekte des Innovationsfonds anhand der oben beschriebenen Ein- und Ausschlusskriterien geprüft. Dabei erfüllten 31 Projekte die Einschlusskriterien und wurden entsprechend in die Datenextraktion eingeschlossen. Die Projekte sind in Tab. A1 (Onlinematerial) den Bundesländern zugeordnet, in denen sie jeweils geleitet wurden.

### Zielgruppen.

Einige Projekte adressierten mehrere Zielgruppen. Am häufigsten wurden Personen in belastenden Lebenssituationen (*n* = 10), Patienten mit Herz-Kreislauf-Erkrankungen (*n* = 5), Patienten mit Erkrankungen der Psyche/Nerven (*n* = 5) und Patienten mit Stoffwechselerkrankungen (*n* = 3) untersucht. Eine genaue Zuordnung der Zielgruppen zu den Kategorien der Lotsenlandkarte des BMC ist in Tab. 1 zu finden. Dort sind auch die in den einzelnen Projekten adressierten Diagnosen nach ICD-10 (5-stellig) zu finden, sofern sie konkret benannt waren.

**Tab. 1:** Adressierte Populationen der eingeschlossenen Projekte, kategorisiert nach Kategorien der Lotsenlandkarte des Bundesverbands Managed Care (BMC; [13]) und Diagnose (nach ICD-10-Codes (5-stellig))

Kategorien der Lotsenlandkarte des BMC	Diagnose nach ICD-10-Codes (5-stellig)	Population/Diagnose laut Projektberichten	Projekte
Geriatrie	Krankheiten der Haut und der Unterhaut	Dekubitus	INVEST- Billstedt/Horn
	Psychische und Verhaltensstörungen	Hirnorganische Erkrankungen (insbesondere Demenz)	NPPV
	Krankheiten des Nervensystems	Morbus Parkinson	NPPV
Herz und Kreislauf	Krankheiten des Kreislaufsystems	Ischämische Herzerkrankungen	INVEST-Billstedt/Horn
		Herzinsuffizienz	INVEST-Billstedt/Horn, MamBo
		Menschen mit (ICD-10) ischämischer Attacke (I63), transitorischer ischämischer Attacke und verwandte Syndrome (G45), intrazerebrale Blutung (I64)	StroCare
		Schlaganfall	NPPV
		Vorhofflimmern	HerzEffektMV
		Therapieresistente Hypertonie	HerzEffektMV
		Akuter Myokardinfarkt	IKK IVP
		Rezidivierender Myokardinfarkt	IKK IVP
		Herzinsuffizienz	IKK IVP
		Subarachnoidalblutung	IKK IVP
		Intrazerebrale Blutung	IKK IVP
		Sonstige nichttraumatische intrakranielle Blutung	IKK IVP
		Hirninfarkt	IKK IVP
		Verschluss und Stenose zerebraler Arterien ohne resultierenden Hirninfarkt	IKK IVP
		Sonstige zerebrovaskuläre Krankheiten	IKK IVP
		Zerebrovaskuläre Störungen bei anderenorts klassifizierten Krankheiten	IKK IVP
		Kardiovaskuläre Erkrankungen (koronare Herzkrankheit, Herzinsuffizienz, Herzrhythmusstörungen)	Cardiolotse
Multimorbidität	Krankheiten des Atmungssystems	Bronchiale Erkrankungen	INVEST-Billstedt/Horn
	Nicht zuzuordnen	Patienten mit mindestens 3 schwerwiegenden chronischen Krankheiten oder Zuständen	MamBo
Muskeln und Skelett	Krankheiten des Muskel-Skelett-Systems und des Bindegewebes	Rheumatoide Arthritis und Psoriasisarthritis	StärkeR
		Erkrankungen der Wirbelsäule	INVEST-Billstedt/Horn
Onkologie	Neubildungen	Bösartige Neubildungen	PIKKO
		Neubildungen	INVEST-Billstedt/Horn
		Onkologische Patienten	OSCAR
Psyche und Nerven	Psychische und Verhaltensstörungen	Psychische Verhaltensstörungen	INVEST-Billstedt/Horn
		Schwere affektive Erkrankungen (insbesondere Depression)	NPPV, RECOVER
		Schizophrene, wahnhafte und bipolare Erkrankungen (Psychosen)	NPPV, RECOVER
		Komplexe Traumafolgestörungen	NPPV, RECOVER
		Zwangsstörung	RECOVER
		Somatische Belastungsstörung	RECOVER
		Essstörung	RECOVER
		Patienten mit komplexen Kommunikationsstörungen	MUK
		Psychisch erkrankte Personen	PREMA
Regionaler Versorgungsbedarf	Nicht zuzuordnen	Patienten des Krankenhauses der Barmherzigen Brüder in Regensburg, die mindestens das Alter von 75 Jahren erreicht hatten	TIGER
		Erwachsene ab 18 Jahren in ländlicher Region mit komplexem oder sektorenübergreifendem Versorgungsbedarf	IGiB-StimMT
Seltene Erkrankungen	Nicht zuzuordnen	Menschen mit seltenen Erkrankungen	TRANSLATE-NAMSE
Stoffwechsel	Krankheiten des Verdauungssystems	Chronisch entzündliche Darmerkrankungen	CED Bio-Assist
	Endokrine, Ernährungs- und Stoffwechselkrankheiten	Diabetes mellitus	INVEST-Billstedt/Horn
	Endokrine, Ernährungs- und Stoffwechselkrankheiten	Adipositas	INVEST-Billstedt/Horn
	Nicht zuzuordnen	Alle Patienten mit Nierentransplantat	NierenTx 360°
Versorgung in belastenden Lebenssituationen	Psychische und Verhaltensstörungen	Familien mit psychosozialen Belastungen durch Erkrankungen der Kinder	KID-PROTEKT
		Menschen mit Demenz und deren pflegende Angehörige	DemStepCare
	Symptome und abnorme klinische und Laborbefunde, die anderenorts nicht klassifiziert sind	Kinder und Jugendliche zwischen 8 und 17 Jahren, die aufgrund von chronischen Schmerzen, einhergehend mit einer schweren Beeinträchtigung der Funktionsfähigkeit, eine interdisziplinäre multimodale Schmerztherapie bekommen	SCHMERZ-NETZ
	Bestimmte Zustände, die ihren Ursprung in der Perinatalperiode haben	Ernährungsprobleme Neugeborener	INVEST-Billstedt/Horn
	Krankheiten des Muskel-Skelett-Systems und des Bindegewebes	Gefährdete und erkrankte Beschäftigte mit Einschränkungen im Bewegungsapparat	BGM-innovativ
	Nicht zuzuordnen	Alte Menschen mit akut sozialpflegerischem Versorgungsbedarf ohne medizinische Indikation für eine Krankenhausbehandlung	GeriNoVe
		Personen < 70 Jahre mit Risiko für Hilfs- und Pflegebedürftigkeit	NWGA
		Erkrankungen während der Schwangerschaft	INVEST-Billstedt/Horn
		Chronisch kranke ältere Menschen (60 Jahre und älter, mind. 3 Hausarztkonsultationen in den letzten 6 Monaten)	HandinHand
		Patienten im Alter von über 70 Jahren (mit akuten oder chronischen (Mehrfach‑)Erkrankungen)	RubiN
		Gesetzlich versicherte Kinder und Jugendliche zwischen 12 und 24 Jahren mit chronischen Krankheiten	TRANSFIT
Sonstige	Nicht zuzuordnen	Faktoren, die den Gesundheitszustand beeinflussen und zur Inanspruchnahme des Gesundheitswesens führen	INVEST-Billstedt/Horn
		Majoramputation der unteren Gliedmaßen	MSTVK
		Menschen mit schlecht heilenden (chronischen) Wunden	VeMaWuRLP

Entsprechend der ICD-10-Klassifizierung kamen Lotsen am häufigsten in der Therapie von Krankheiten des Kreislaufsystems (*n* = 18) und von psychischen und Verhaltensstörungen (*n* = 12) zum Einsatz. Nicht in allen Fällen war die untersuchte Population einer exakten Erkrankung zuzuordnen. So wurde in 3 Projekten der Einsatz von Lotsen in der Versorgung von älteren Menschen ohne vorab definierte Diagnose untersucht. Ein Projekt untersuchte zudem Personen mit einem Risiko, chronisch zu erkranken, 2 adressierten Menschen, die mit einem komplexen oder akut sozialpflegerischen Versorgungsbedarf im ländlichen Raum leben.

### Aufgaben der Lotsen.

In Tab. 2 sind die Aufgaben, orientiert an Kelly et al. [16], dargestellt, denen die Lotsen in den einzelnen Projekten nachgegangen sind. In 29 Projekten koordinierten die Lotsen die Versorgung zwischen verschiedenen Leistungserbringenden und in 30 Projekten wurde von ihnen auch das Fall-Monitoring und/oder die Bedarfserhebung vorgenommen. Information, Schulung und/oder Beratung der Patienten leisteten die Lotsen in 25 Projekten. Zu Angeboten in Ergänzung zur Versorgung (z. B. Selbsthilfegruppen, Bewegungsangebote) wurde in 20 Projekten von Lotsen navigiert. Seltener übernahmen die Lotsen explizit administrative Aufgaben für die Patienten (*n* = 10), wie zum Beispiel die Beantragung einer Rehamaßnahme, leisteten psychosoziale Unterstützung (*n* = 9) oder waren am Aufbau regionaler Angebote (*n* = 4) beteiligt. Teilweise wurden einige Aufgaben in andere Komponenten der Neuen Versorgungsformen integriert, wie zum Beispiel ins Aufgabenprofil der Netzwerk- und Quartiersmanager (NWGA) oder ein neu geschaffenes Angebot zur Stressreduktion (INVEST-Billstedt/Horn).

**Tab. 2:** Aufgaben der Lotsen innerhalb der Projekte, kategorisiert nach Kelly et al. [16]

Aufgabe	Projekte
Koordination der Versorgung (Überweisungen, Entlassung, Reha)	DemStepCare, IGiB-StimMT, MUK, TRANSLATE-NAMSE, IKK IVP, NierenTx 360°, TIGER, STROKE OWL, CED Bio-Assist, GeriNoVe, NWGA, SCHMERZ-NETZ, PIKKO, StärkeR, Familien-SCOUT, INVEST – Billstedt/Horn, StroCare, NPPV, RECOVER, MamBo, HerzEffektMV, MSTVK, VeMaWuRLP, BGM-innovativ, HandinHand, OSCAR, RubiN, TransFIT, Cardiolotse
Fall-Monitoring und/oder Erhebung der Bedarfe der Patienten	DemStepCare, IGiB-StimMT, MUK, TRANSLATE-NAMSE, IKK IVP, NierenTx 360°, TIGER, STROKE OWL, CED Bio-Assist, GeriNoVe, NWGA, SCHMERZ-NETZ, PIKKO, StärkeR, Familien-SCOUT, INVEST – Billstedt/Horn, StroCare, RECOVER, MamBo, HerzEffektMV, MSTVK, VeMaWuRLP, BGM-innovativ, HandinHand, OSCAR, RubiN, TransFIT, Cardiolotse, PREMA
Aufbau regionaler Angebote („community engagement“)	NierenTx 360°, Familien-SCOUT, VeMaWuRLP, RubiN
Information, Schulung und/oder Beratung	DemStepCare, MUK, PREMA, TRANSLATE-NAMSE, TIGER, STROKE OWL, CED Bio-Assist, GeriNoVe, KID-PROTEKT, NWGA, SCHMERZ-NETZ, PIKKO, StärkeR, Familien-SCOUT, INVEST-Billstedt/Horn, StroCare, NPPV, MamBo, HerzEffektMV, MSTVK, VeMaWuRLP, HandinHand, OSCAR, TransFIT, Cardiolotse
Übernahme von/Unterstützung bei administrativen Aufgaben	MUK, TIGER, GeriNoVe, RECOVER, MamBo, MSTVK, BGM-innovativ, HandinHand, OSCAR, RubiN
Psychosoziale Unterstützung	DemStepCare, TRANSLATE-NAMSE, TIGER, CED Bio-Assist, SCHMERZ-NETZ, Familien-SCOUT, RECOVER, HerzEffektMV, OSCAR
Navigation von Angeboten in Ergänzung zur Versorgung	IGiB-StimMT, IKK IVP, NierenTx 360°, TIGER, STROKE OWL, CED Bio-Assist, GeriNoVe, KID-PROTEKT, NWGA, PIKKO, Familien-SCOUT, NPPV, RECOVER, MamBo, MSTVK, BGM-innovativ, OSCAR, RubiN, TransFIT, Cardiolotse

### Qualifikationen.

In Tab. A2 (Onlinematerial) ist die Berufsgruppenzugehörigkeit der Lotsen in den einzelnen Projekten dargestellt. Über alle Projekte hinweg gehörten die Lotsen am häufigsten (*n* = 19) der Berufsgruppe der examinierten Pflegefachkräfte an, gefolgt von der Berufsgruppe der Sozialarbeiter oder Sozialpädagogen (*n* = 9) und der Gruppe der medizinischen Fachangestellten (*n* = 8). Auch Therapeuten (*n* = 5), Ärzte (*n* = 2), Gerontologen (*n* = 1) und Sozialversicherungsangestellte/Krankenkassenfachwirte (*n* = 1) wurden als Lotsen eingesetzt. In einem Projekt ging die Berufsgruppenzugehörigkeit nicht aus dem Ergebnis- oder Evaluationsbericht hervor (NPPV).

### Rechtsgrundlage.

In Tab. A3 (Onlinematerial) ist die Rechtsgrundlage für den Einsatz der Lotsen innerhalb der Projekte dargestellt. Am häufigsten (*n* = 23) bildete ein Selektivvertrag nach § 140a SGB V die Rechtsgrundlage für die Neue Versorgungsform, gegebenenfalls ergänzt um einen Behandlungsvertrag nach § 630a BGB, der teilweise auch die ausschließliche Rechtsgrundlage war. Zwei Projekte waren Modellvorhaben nach § 63, 64 Abs. 1 SGB V. In 3 weiteren Projekten gab es individuelle Lösungen für die Rechtsgrundlage. Aus 3 Projektberichten ging die Rechtsgrundlage der Neuen Versorgungsform nicht hervor.

### Finanzierung.

Nicht immer wurden die Lotsen über Projektgelder finanziert. In einem Projekt wurden die Lotsen zum Beispiel aus den Eigenmitteln eines Krankenhauses bezahlt (TIGER), in einem weiteren Projekt wurden nach Projektende 2 Lotsen in einem Krankenhaus aus Eigenmitteln weiterfinanziert (PIKKO). Im Projekt BGM-innovativ waren die Lotsen direkt bei der Betriebskrankenkasse angestellt.

### Studiencharakteristika.

In Tab. 3 sind die Studiencharakteristika der Evaluationskonzepte für die einzelnen Projekte und deren Fördersumme gemeinsam mit einer Kurzbeschreibung der Neuen Versorgungsform und der Transferempfehlung des Innovationsausschusses dargestellt. In 11 Projekten kamen zur Evaluation randomisierte kontrollierte Studien (RCTs) zum Einsatz, 3 davon waren Cluster-randomisiert. In 17 Projekten wurde eine quasiexperimentelle Interventionsstudie durchgeführt, d. h., es gab prospektiv rekrutierte Interventions- und Kontrollgruppen, die nicht randomisiert gebildet wurden. In 4 Projekten wurde eine Kohorte vor und nach Etablierung einer Intervention untersucht, ohne dass es eine Kontrollgruppe gab (einarmige prospektive Kohortenstudie). In 3 Projekten wurden zusätzlich qualitative Methoden in der summativen Evaluation eingesetzt, um vertiefte Einblicke in quantitative Ergebnisse zu erhalten (sog. sequenzielle, explizierende Mixed-Methods-Designs; GeriNoVe, MSTVK, IGiB-StimMT).

**Tab. 3:** Übersicht der eingeschlossenen Projekte

Projekt	Fördersumme (in Millionen)	Neue Versorgungsform und Setting (Lotsen hervorgehoben)	Studiendesign	Errechnete/realisierte Stichprobengröße (letztes Follow-up)	Transferempfehlung des Innovationsausschusses
BGM-innovativ	3,74	*Fallmanager* bei Betriebskrankenkasse	RCT (individuelle Randomisierung)	350/729	Keine Empfehlung/Weitergabe der wissenschaftlichen Erkenntnisse
Cardiolotse	4,6	*Cardiolotse* an kommunalen Krankenhäusern	RCT (individuelle Randomisierung)	2908/2550	Keine Empfehlung/Weitergabe der wissenschaftlichen Erkenntnisse
CED Bio-Assist	5,4	*Lotsenfunktion* bei niedergelassenen Gastroenterologen	RCT (individuelle Randomisierung)	1054/1086	Keine Empfehlung
DemStepCare	4,4	*Case-Management* und Krisenambulanzdienst	RCT (Cluster-Randomisierung)	960/130	Keine Empfehlung
Familien-SCOUT	2,8	*Comprehensive Care und Case Management*, angestellt bei Verband oder Universitätsklinikum	Quasiexperimentelle Interventionsstudie	358/474	Prüfung/Überführung
GeriNoVe	4,5	Pflegegeleitetes „Regionales geriatrisches Notfall-Versorgungszentrum“ mit Pflegefachkräften und *Case-Management*	Prospektive Kohortenstudie (einarmig)	1024/592	Keine Empfehlung
HandinHand	8	*Pflegeexperten* an einem koordinierenden Zentrum	Prospektive Kohortenstudie (einarmig)	517/712	Keine Empfehlung/Weitergabe der wissenschaftlichen Erkenntnisse
HerzEffektMV	10	*Fallmanager* am Care Center (Universitätsklinikum)	RCT (individuelle Randomisierung)	890/957	Keine Empfehlung
IGiB-StimMT	14,5	*Case-Management* an einem neu aufgebauten ambulanten Koordinierungs- und Beratungszentrum	Quasiexperimentelle Interventionsstudie	–/10.613	Prüfung/Überführung
IKK IVP	3,5	*Patientenkoordinatoren* der Krankenkasse, bei Bedarf Pflegeberatung	Quasiexperimentelle Interventionsstudie	19.055/19.055	Keine Empfehlung
INVEST-Billstedt/Horn (gesundheitliche Chancen)	6,3	Gesundheitskiosk, *Community Health Nurse*	Prospektive Kohortenstudie (einarmig)	–/1452	Prüfung/Überführung
INVEST-Billstedt/Horn (Gesundheitsökonomie)	–	Netzwerkmanager		–/53.133	
INVEST-Billstedt/Horn (Patientenorientierung)	–	Indikations- und zielgruppenspezifische *Versorgungskoordinatoren*		–/98	
KID-PROTEKT	2,2	*Lotsen* für das Schnittstellenmanagement zwischen Arztpraxis (Frauen- bzw. Kinderheilkunde) und Jugendhilfe	Quasiexperimentelle Interventionsstudie	179/527	Keine Empfehlung
MamBo (Gesundheitsökonomie)	3,4	*Monitoring- und Koordinationsassistentinnen* übernehmen Case-Management (Gesundheitsnetz), Bedarfsmanagement (Krankenkassen), Versorgungsmanagement (Gesundheitsnetz)	Quasiexperimentelle Interventionsstudie	–/4114	Keine Empfehlung
MamBo (Patientenbefragung)	–		Quasiexperimentelle Interventionsstudie	459/354	
MUK	2,17	*Case-Management *an einem Universitätsklinikum, Patiententraining, „UK-Therapie“	Quasiexperimentelle Interventionsstudie	408/172	Keine Empfehlung
MSTVK	0,38	*Care-Management* an einem Universitätsklinikum	Quasiexperimentelle Interventionsstudie	70/63	Keine Empfehlung
NierenTx 360°	6	*Fallmanagement *am Nierentransplantationszentrum, telemedizinische Nachuntersuchungen, psychosomatisch-psychosoziale Risiko-Assessments, telemedizinische Adhärenz-Coachings, kardiovaskuläre Assessments, telemedizinisch begleitete Trainingstherapie	Quasiexperimentelle Interventionsstudie	1533/1009	Keine Empfehlung
NPPV	13	Ambulanter Bezugsarzt/-therapeut, Netzwerkmanager, *Case-Manager*	Quasiexperimentelle Interventionsstudie	2912/6709	Keine Empfehlung/Weitergabe der wissenschaftlichen Erkenntnisse
NWGA	8,9	*Fallmanagement* im Netzwerk	Quasiexperimentelle Interventionsstudie	3000/2670	Keine Empfehlung
		Quartiermanagement			
		Schulungsveranstaltungen			
OSCAR	1,4	*Social Care Nurse* an Krankenhäusern	Quasiexperimentelle Interventionsstudie	350/362	Keine Empfehlung/Weitergabe der wissenschaftlichen Erkenntnisse
PIKKO	3,6	Onko-Lotsen bei teilnehmenden ambulanten und stationären Leistungserbringenden, psychosoziale Beratungsleistungen der Krebsberatungsstellen, onkologische Wissensdatenbank	Quasiexperimentelle Interventionsstudie	1014/1004	Keine Empfehlung
PREMA	4,9	*Case-Management* im hausärztlichen Setting	RCT (Cluster-Randomisierung)	–/21	Keine Empfehlung
		Online-Plattform (mit u. a. psychoedukativen Videos und Texten, Selbsthilfeübungen)			
RECOVER	6,8	Gestuftes, integriertes und koordiniertes Versorgungsmodell	RCT (individuelle Randomisierung)	890/317	Keine Empfehlung/Weitergabe der wissenschaftlichen Erkenntnisse
		*Case-Management* als ein Element für Personen mit hohem Schweregrad (Stufe 3)			
RubiN	8,1	*Case-Management* angestellt bei Praxisnetzen	Quasiexperimentelle Interventionsstudie	4400/4489	Keine Empfehlung
SCHMERZ-NETZ	1,3	Multiprofessionelle Nachsorgeteams aus *Sozialarbeitenden,* Ärzten, Psychologen an Kliniken	RCT (individuelle Randomisierung)	394/419	Prüfung/Überführung
StärkeR	2,1	*Rheumatologische Fachassistenz* bei Praxen und Krankenhausambulanzen	RCT (individuelle Randomisierung)	800/601	Keine Empfehlung/Weitergabe der wissenschaftlichen Erkenntnisse
StroCare	3,8	Schlaganfallexperten (Neurologen und *Stroke Nurses*) in Akutkliniken unterstützt durch *Case-Management *(Krankenkasse)	Quasiexperimentelle Interventionsstudie	235/406	Keine Empfehlung
STROKE OWL	7,1	*Schlaganfall-Lotsen* an Akutkrankenhäusern	Quasiexperimentelle Interventionsstudie	2538/2334	Keine Empfehlung
TIGER	3,7	*Pfadfinder* am Krankenhaus	RCT (individuelle Randomisierung)	280/244	Keine Empfehlung
TransFIT	1,6	*Fallmanagement* an Kliniken	Quasiexperimentelle Interventionsstudie	1890/1440	Keine Empfehlung
TRANSLATE-NAMSE	13,4	*Lotsen und ärztliche Koordinatoren* an Zentren für seltene Erkrankungen, interdisziplinäre Fallkonferenzen	Prospektive Kohortenstudie (einarmig)	9707/9707	Prüfung/Überführung
VeMaWuRLP	1,9	*Fallmanager* (beauftragt durch eine Managementgesellschaft)	RCT (individuelle Randomisierung)	300/186	Keine Empfehlung

*RCT* randomisierte kontrollierte Studie

In 4 Projekten (13 %) wurde insgesamt oder für Teilfragestellungen keine Poweranalyse zur Errechnung der benötigten Stichprobengröße berichtet. Innerhalb der Studien, deren Effektmessung nicht ausschließlich auf Routinedaten beruhte, reichte die bis einschließlich letztes Follow-up realisierte Fallzahl von 21 bis 6709 Teilnehmenden. 15 Projekte mit errechneter Stichprobengröße (48 %) erreichten diese beim letzten Follow-up nicht, wobei der Anteil fehlender Fälle zum letzten Follow-up zwischen 1 % (PIKKO) und 86 % (DemStepCare) variiert.

### Outcomes.

Insgesamt wurden in den 31 eingeschlossenen Projekten 153 verschiedene Outcomes gemessen (siehe Tab. A4, Onlinematerial). Diese lassen sich den Outcome-Domänen Patient-reported Outcome (PROMs, *n* = 28) und Patient-reported Experience Measures (PREMs, *n* = 9), Prozessindikatoren (*n* = 51), gesundheitsökonomische Indikatoren (*n* = 16), klinische Outcomes (*n* = 38) und Beschreibungen der patientenbezogenen Versorgungssituation (z. B. Pflegebedürftigkeit der Patienten; *n* = 11) zuordnen. Das am häufigsten gemessene PROM war Lebensqualität, die 21-mal als primärer oder sekundärer Endpunkt, bezogen auf eine spezielle Krankheitslast, physisch, psychisch oder allgemein, gemessen wurde. Therapiezufriedenheit wurde 7‑mal als PREM gemessen. Prozessindikatoren beziehen sich hauptsächlich auf die Inanspruchnahme von Versorgungsleistungen, wobei der Schwerpunkt auf der stationären Versorgung lag (*n* = 21). Gesundheitsökonomische Indikatoren beziehen sich vor allem auf Kosten, berichtet entweder als Gesamtkosten der Neuen Versorgungsform (*n* = 7) oder u. a. differenziert nach GKV-Leistungsausgaben (*n* = 6), anfallenden Kosten im ambulanten (*n* = 5) bzw. stationären Versorgungssektor (*n* = 5), Kosten für Arzneimittel (*n* = 4), Heil‑/Hilfsmittel (*n* = 1) und Pflege (*n* = 1) sowie Kosten, die durch Arbeitsausfall entstehen (*n* = 7). Weitere gesundheitsökonomische Indikatoren sind Kosten, die bei dem Patienten anfallen (*n* = 2) und Kosteneffektivität (*n* = 12). Klinische Outcomes unterscheiden sich aufgrund des heterogenen Indikationsspektrums stark zwischen den Projekten, sodass lediglich Depressivität (*n* = 9), Angst/-zustände (*n* = 7), Mortalität (*n* = 5), physische Funktionsfähigkeit (*n* = 2) und Schlaganfallrezidive (*n* = 2) in mehr als einem Projekt gemessen wurden. Bezogen auf die patientenbezogene Versorgungssituation wurden Pflege- und Hilfebedürftigkeit in 4 Projekten analysiert. 4-mal wurde die Belastung für Angehörige, die Familie oder Eltern von einer Erkrankung betroffener Personen, 3‑mal die Verfügbarkeit sozialer oder Peer-Unterstützung untersucht.

### Effekte der Lotsenprojekte.

**Abb. 1:**
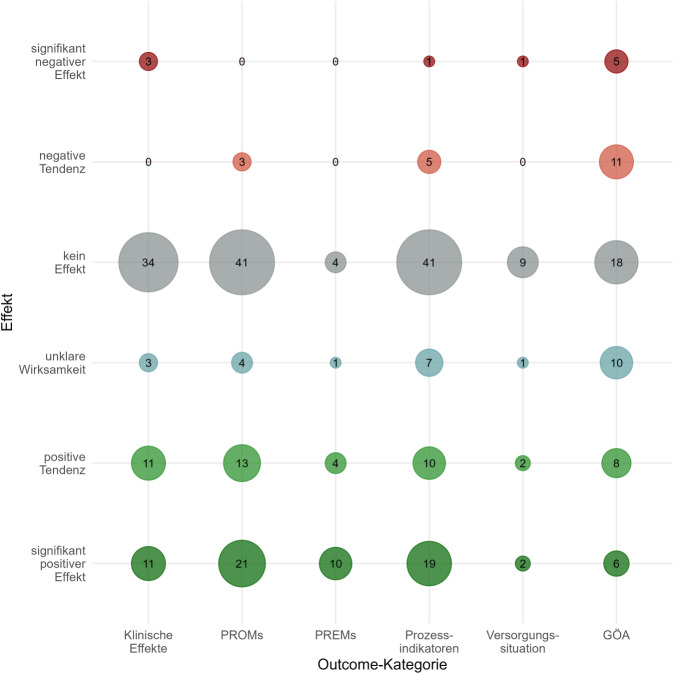
Ergebnisübersicht der Lotsenprojekte (primäre und sekundäre Endpunkte, *GÖA* Outcomes gesundheitsökonomischer Analysen)

Abb. 1 fasst die Ergebnisse der primären und sekundären Endpunkte differenziert nach Outcome-Kategorie grafisch zusammen. Abgesehen von PREMs zeigte sich in allen Outcome-Kategorien am häufigsten kein Effekt. In Summe war 147-mal kein Effekt auf das primäre oder sekundäre Outcome messbar. Signifikant positive Effekte wurden am häufigsten für PROMs (21-mal), Prozessindikatoren (19-mal) und PREMs (10-mal) gemessen. Die Lotsenintervention hatte demnach in 8 Projekten (26 %) einen signifikant positiven Effekt auf die Lebensqualität der Patienten, in 16 Projekten (52 %) war allerdings kein Effekt aufzuzeigen.

In 6 Projekten wurde ein signifikant positiver Effekt der Intervention auf Hospitalisierungsraten und Verweildauern im Krankenhaus gemessen, in 8 Projekten kein Effekt. In 6 Projekten wurde ein signifikant positiver Effekt der Intervention auf die Zufriedenheit der Patienten mit ihrer Versorgung, Therapie oder den ihnen zur Verfügung stehenden Hilfsmitteln gemessen, 1‑mal war die Wirksamkeit unklar.

Am häufigsten über alle Outcome-Domänen hinweg (5-mal) fanden sich signifikant negative Effekte in den gesundheitsökonomischen Outcomes (GÖA) im Sinne einer Kostensteigerung. 6‑mal konnte ein signifikant positiver Effekt nachgewiesen werden.

Signifikant positive klinische Effekte zeigten sich am häufigsten (*n* = 3) auf die Depressivität der untersuchten Patienten. In ebenso vielen Projekten zeigten sich keine Effekte auf Depressivität, 1‑mal zudem eine signifikante Verschlechterung des depressiven Zustands.

**Abb. 2:**
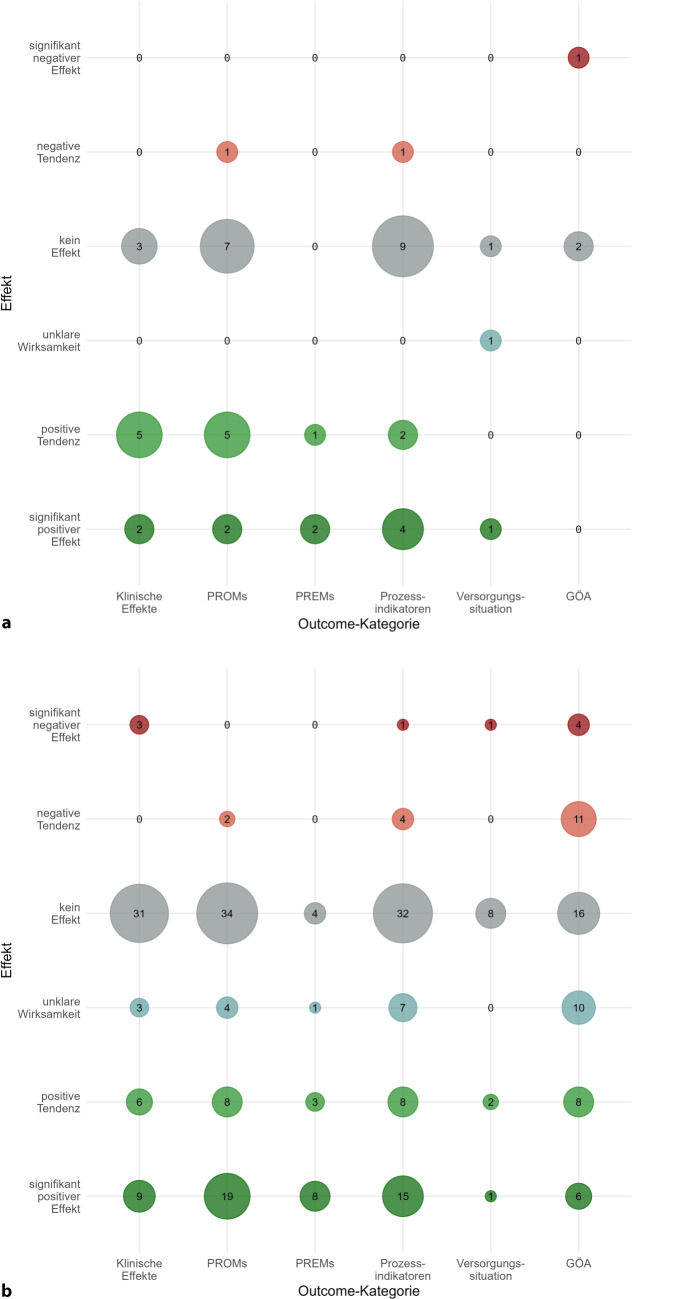
Ergebnisübersicht **a** der primären Endpunkte; **b** der sekundären Endpunkte

Waren klinische Endpunkte das primäre Outcome eines Projekts, so überwogen Projekte, die positive Effekte (signifikante und tendenzielle) gemessen haben, gegenüber denjenigen, die keinen Effekt zeigen konnten (Abb. 2a). Dasselbe gilt für die Outcome-Domäne der PREMs, wo ausschließlich positive Effekte auf das primäre Outcome gemessen wurden. In der Domäne der PROMs halten sich positive und keine Effekte auf das primäre Outcome die Waage. Für Prozessindikatoren als primäres Outcome überwiegen Projekte, die keinen (signifikanten) Effekt messen konnten, gegenüber solchen, die einen positiven Effekt nachweisen konnten, ebenso wie bzgl. der patientenbezogenen Versorgungssituation. Waren gesundheitsökonomische Indikatoren das primäre Outcome, zeigte sich in keinem Projekt ein positiver Effekt und 1‑mal ein signifikant negativer im Sinne einer Kostensteigerung.

Abgesehen von den PREMs, wo insgesamt 11-mal ein tendenziell oder signifikant positiver Effekt gemessen werden konnte, überwiegen für die sekundären Outcomes in allen Domänen Projekte, die keine oder signifikant bzw. tendenziell negative Effekte zeigten (Abb. 2b). Für gesundheitsökonomische Indikatoren als sekundäres Outcome war 10-mal keine Effektmessung möglich, weil etwa keine Kontrollgruppe oder zu wenige Probanden verfügbar waren.

In Tab. A5 (Onlinematerial) sind positive, negative, keine und unklare Effekte bezogen auf die Zielgruppen dargestellt. Dabei wurden signifikant positive und negative Effekte jeweils mit den entsprechenden Tendenzen zusammengefasst. In den Kategorien „Krankheiten des Kreislaufsystems“, „Multimorbidität“, „Krankheiten des Muskel-Skelett-Systems und des Bindegewebes“ und „Neubildungen“ sind für ein besseres Verständnis mehrere Projekte zusammengefasst. Die übrigen Kategorien sind nur durch jeweils ein Projekt vertreten. Für Patienten mit Nierentransplantat, alte Menschen mit akutem sozialpflegerischem Versorgungsbedarf, Familien mit psychosozialen Belastungen durch Erkrankungen der Kinder, Patienten mit Symptomen und abnormen klinischen und Laborbefunden, die anderenorts nicht klassifiziert sind, sowie für Patienten mit Neubildungen überwogen die positiven gegenüber den negativen oder keinen bzw. unklaren Effekten, für Patienten mit schlecht heilenden (chronischen) Wunden hielten sich die Effekte die Waage. Für alle anderen Zielgruppen überwogen negative, unklare oder keine Effekte gegenüber den positiven.

**Abb. 3:**
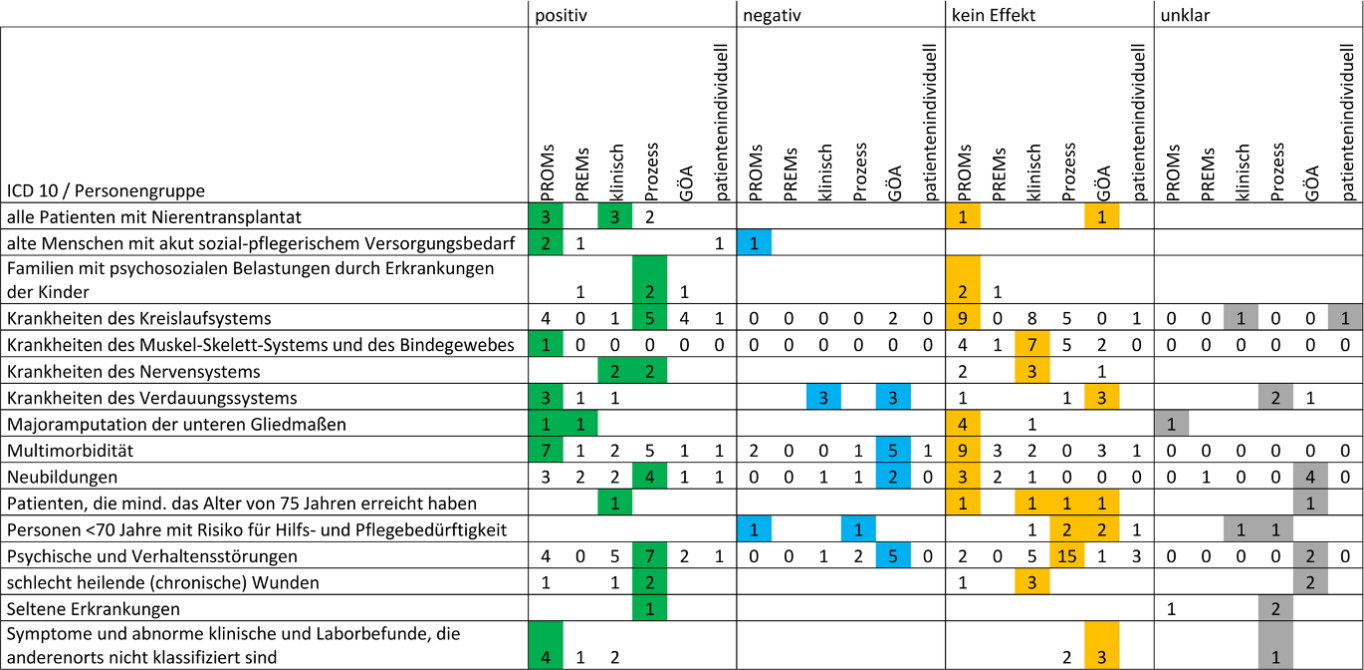
Anzahl verschiedener Outcomes differenziert nach ICD-10 bzw. Personengruppe. Farblich hervorgehoben sind die jeweils am häufigsten gemessenen Effekte. (*GÖA* Outcomes gesundheitsökonomischer Analysen)

Positive Effekte auf klinische Outcomes (tendenziell und signifikant) wurden mehr als einmal für Patienten mit Nierentransplantat, Krankheiten des Nervensystems, multimorbide Patienten, Patienten mit Neubildungen, Patienten mit psychischen und Verhaltensstörungen und für Patienten mit Symptomen und abnormen klinischen und Laborbefunden, die anderenorts nicht klassifiziert sind, erzielt (Abb. 3). Für alle Zielgruppen überwiegen positive Effekte auf PROMs, PREMs und Prozessindikatoren. Negative Effekte auf klinische Outcomes zeigen sich vor allem bei Krankheiten des Verdauungssystems. Bei Krankheiten des Muskel-Skelett-Systems und des Bindegewebes zeigte sich am häufigsten kein Effekt auf klinische Outcomes. Positive Effekte auf PROMs zeigen sich am häufigsten für multimorbide Patienten und solche mit Symptomen und abnormen klinischen und Laborbefunden, die anderenorts nicht klassifiziert sind. Positive Effekte auf PREMs zeigen sich am häufigsten für Patienten mit Neubildungen. Prozessindikatoren können durch den Einsatz von Lotsen vor allem für Patienten mit psychischen und Verhaltensstörungen verbessert werden. Für 15 Outcomes zeigten sich in dieser Zielgruppe keine Effekte auf Prozessindikatoren. Gesundheitsökonomische Analysen zeigen Anzeichen für Kostensenkungen durch den Einsatz vor allem für Patienten mit Krankheiten des Kreislaufsystems. Details sind in Abb. 3 dargestellt.

Die Empfehlungen des Innovationsausschusses zu den einzelnen Projekten sind in Tab. 3 dargestellt. Von den 31 eingeschlossenen Projekten haben 26 keine Empfehlung zur Überführung in die Regelversorgung erhalten. Bei 19 Projekten wurden auch keine Ergebnisse an entsprechende Akteure weitergeleitet. Bei 7 der 26 Projekte ohne Empfehlung des Innovationsausschusses wurden die wissenschaftlichen Erkenntnisse zur Information unter anderem an Akteure der Selbstverwaltung weitergeleitet und bei 5 Projekten wird eine Überführung in die Regelversorgung durch den Innovationsausschuss empfohlen und durch die Adressaten der weitergeleiteten Ergebnisse geprüft.

## Diskussion

Im vorliegenden Review wurden 31 Projekte aus der Förderlinie Neue Versorgungsformen des Innovationsfonds, in denen der Einsatz von Lotsen in der medizinischen Versorgung untersucht wurde, hinsichtlich Zielgruppe, Aufgaben, Qualifikationen, Rechtsgrundlage, Studiencharakteristika und Outcomes analysiert. Der hohe Anteil an Lotsenprojekten an den Neuen Versorgungsformen (*n* = 31/99) deutet auf ein hohes Problembewusstsein und eine positive Erwartungshaltung gegenüber dem Einsatz von Lotsen im Gesundheitssystem seitens der Akteure im Innovationsausschuss hin.

So heterogen wie die Aufgaben der Lotsen und die gewählten Outcomes, anhand derer Effektivität gemessen werden sollte, waren auch die erzielten Effekte. So spiegeln 147 Outcomes (wenn auch teilweise zu mehreren Messzeitpunkten), für die über alle 31 Projekte hinweg kein Effekt gemessen wurde, zwar einerseits eine weitestgehend unklare Wirksamkeit des Einsatzes von Lotsen im Rahmen der Projekte wider, sprechen andererseits aber auch für einen von vornherein unklaren Wirkmechanismus. Theorien, Modelle oder der Einsatz qualitativer Methoden im Vorfeld der summativen Evaluation können helfen, Wirkmodelle zu entwickeln, und ermöglichen außerdem auf lange Sicht eine Festlegung auf Outcomes, die, im Sinne eines Core-Outcome-Sets, für die Evaluation vergleichbarer Interventionen bedeutsam sind [17–19]. Bis auf wenige Ausnahmen (z. B. SCHMERZ-NETZ) war nicht eindeutig zu erkennen, welche Annahmen zur Wirkung der Lotsen die Entwickler oder Evaluatoren der Neuen Versorgungsform zugrunde gelegt haben [17].

Erste Schlüsse bzgl. relevanter Outcomes lassen die Ergebnisse des vorliegenden Reviews durchaus zu, zeigten sich doch Effekte vor allem in den Domänen PROMs und Prozessindikatoren. Insbesondere eine Reduktion der Inanspruchnahme ambulanter und stationärer Leistungen, eine gesteigerte Therapiezufriedenheit und Lebensqualität seitens der untersuchten Zielgruppen waren für chronisch an Herz oder Kreislauf erkrankte, multimorbide und psychisch kranke Patienten durchaus zu beobachten. Ähnliche Effekte zeigten auch 2 internationale Umbrella-Reviews zur Navigation onkologischer Patienten sowie Gaertner und Kollegen in Bezug auf Case-Management für Personen ab 65 Jahren in Deutschland [20–22]. Positive Effekte auf Depressivität konnten auch Smith et al. nachweisen [23].

Ob diese Effekte als Mediatoren für eine Wirksamkeit von Lotsen auf klinische Outcomes wirken können oder ob ein direkter Effekt erwartet werden kann, müsste Gegenstand eines Wirkmodells sein [24]. Dabei wäre auch zu beachten, dass patientenberichtete Endpunkte insbesondere zur Zufriedenheit mit der Therapie auch der sozialen Erwünschtheit (Response Bias) unterliegen können [25]. Zudem birgt die unterschiedliche Anzahl an gemessenen Outcomes je Projekt ein Verzerrungsrisiko, möglicherweise auch in den Augen des Innovationsausschusses, da Projekte mit vielen sekundären Outcomes – für die zudem keine Poweranalyse erfolgte – allein deshalb häufiger positive ebenso wie negative Effekte zeigen können. Eine differenzierte Betrachtung der Effekte für primäre und sekundäre Endpunkte (Abb. 2) zeigt deutlich, dass die Fülle an nicht messbaren Effekten vor allem auf die sekundären Endpunkte zurückzuführen ist. Waren klinische Outcomes als primäre Outcomes gesetzt, so ließen sich zumindest tendenziell positive Effekte des Lotseneinsatzes feststellen, vor allem für Patienten mit psychischen und Verhaltensstörungen.

Neben einheitlich über alle Projekte hinweg gemessenen Outcomes, die so auch für Studien zur Wirksamkeit von Case-Management fehlen [22], ist für die Beurteilung eines Gesamteffekts der Lotsen eine Vergleichbarkeit der Intervention eine zentrale Voraussetzung. Wie für komplexe Interventionen bereits häufiger beobachtet, ist diese Voraussetzung auch in den vorliegenden Projekten nicht erfüllt [26, 27]. Zu heterogen sind die Aufgaben und Einsatzgebiete der Lotsen. Aufgrund der unterschiedlichen Settings, in denen Lotsen eingesetzt werden, und der verschiedenen Zielgruppen, die sie adressieren, ist eine solche Standardisierung der Intervention schwer vorstellbar. Für eine dennoch notwendige einheitliche Bewertung wären daher einheitliche Reporting-Guidelines, z. B. nach dem Vorbild digitaler Interventionen, sinnvoll [28], die Vorgaben für das einheitliche Berichten von Interventionskomponenten, Charakteristika der Zielgruppe und gemessenen Effekten machen. Um ähnlich gelagerte Lotseninterventionen zu vergleichen, könnten auf Basis der hier vorgestellten Ergebnisse weiterführende Subgruppenanalysen durchgeführt werden (z. B. Altersgruppen, Schweregrad der Erkrankung, Intensität der Lotsenbetreuung). In Projekten, die einen regionalen Versorgungsbedarf adressieren, wie etwa INVEST-Billstedt/Horn, sind Lotsen zudem Teil größerer Interventionen (im konkreten Fall eines Gesundheitskiosks), sodass Effekte schwer auf die Lotsen als einzelne Interventionskomponente zurückzuführen sind. Dieser Rückschluss wird in einzelnen Projekten zudem dadurch erschwert, dass Lotsen aufgrund ihrer Berufsgruppenzugehörigkeit vermutlich noch Aufgaben übernehmen, die in den Projektberichten nicht nachvollziehbar sind.

Eine weitere Limitation der betrachteten Analysen ergibt sich aus den festgestellten unklaren Effekten u. a. aufgrund zum letzten Follow-up zu geringer Fallzahlen und des damit verbundenen Risikos falsch-negativer Studienergebnisse. Letzteres kann bei einem Loss-to-Follow-up von durchschnittlich 30 %, der damit über dem Schwellenwert der evidenzbasierten Medizin (EBM) für Low-quality Studies liegt, nicht verwundern [29]. Hierzu ist allerdings zu bemerken, dass in einigen Projekten der Interventionszeitraum zumindest teilweise in die COVID-19-Pandemie fiel, was Schwierigkeiten bei der Rekrutierung und Interventionstreue mit sich gebracht haben könnte. Zusammengenommen erschweren die bisher beschriebenen methodischen Schwächen der Projekte eine notwendige Replikation der Ergebnisse in bisher nicht adressierten Settings. Ein Austausch zwischen den geförderten Projekten könnte helfen, anhand der in der jeweiligen Evaluation gemachten Erfahrungen einige der genannten methodischen Probleme zu adressieren und Evaluationsergebnisse projektübergreifend zu diskutieren.

Aus ökonomischer Perspektive sind die teils signifikant negativen Effekte auf gesundheitsökonomische Indikatoren auffällig, da sie für eine Kostensteigerung durch den Einsatz von Lotsen oder deren mangelnde Kosteneffektivität sprechen. Zu einem ähnlichen Ergebnis kam auch das Review von Brinkmann et al. zur Kosteneffektivität von Case- und Care-Management bei älteren Populationen [26], während Hawkins und Kollegen durchaus Kosteneinsparungen durch Case-Management für Hochrisikopatienten, die in den USA Medicare beziehen, feststellten [30]. Angesichts der Erkenntnisse aus Deutschland sollte der nicht geringe Mitteleinsatz für die untersuchten Projekte (Tab. 3) trotz der breiten Palette an Einsatzgebieten für Lotsen kritisch geprüft werden. Auch für die Überführung in die Regelversorgung und die dafür notwendige Vergütungs- und Finanzierungsstruktur der Lotsentätigkeiten, für die bereits verschiedene rechtliche Verankerungen diskutiert werden, sollte die verfügbare Evidenz der Modellprojekte zur Kosteneffektivität berücksichtigt werden [31]. Alternativ könnten längere Förderzeiträume in Erwägung gezogen werden, um auch langfristige ökonomische Effekte messen zu können [32].

Ein Großteil der Projekte (*n* = 26) erhielt keine Transferempfehlung des Innovationsausschusses, auch wenn bei 7 der 26 Projekte die wissenschaftlichen Erkenntnisse u. a. an Akteure der Selbstverwaltung weitergeleitet wurden. Für 5 weitere Projekte (Familien-SCOUT, IGiB-StimMT, INVEST Billstedt/Horn, SCHMERZ-NETZ und TRANSLATE-NAMSE) hat der Innovationsausschuss eine Empfehlung ausgesprochen. Deren detaillierte Ergebnisse sind in Tab. A6 (Onlinematerial) dargestellt. Die adressierten Akteure, denen die erzielten Projektergebnisse weitergeleitet wurden, wurden aufgefordert, Finanzierungsmöglichkeiten und Erweiterungen bestehender Rechtsgrundlagen zu prüfen. Unter anderen diskutieren die entsprechenden Akteure daraufhin, dass Lotsen mitunter auch Aufgaben übernehmen, die nicht in das Leistungsspektrum der gesetzlichen Krankenversicherung fallen, bereits Bestandteil des Diskurses auf Bundesebene oder Teil der Regelversorgung sind. Der Einsatz von Lotsen birgt damit die Gefahr, Doppelstrukturen zu implementieren und den Fachkräftemangel zu verschärfen. Letzteres gilt vor allem, weil die Lotsenfunktion am häufigsten von examiniertem Pflegepersonal übernommen wird, wie die untersuchten Projekte zeigten, und dieses Pflegepersonal dann ggf. an anderer Stelle fehlt [1]. Diese Überlegungen sollten der positiven Erwartungshaltung des Innovationsausschusses, die in den vielen geförderten Lotsenprojekten zum Ausdruck kommt, ebenso wie den entsprechenden Empfehlungen des Sachverständigenrats Gesundheit & Pflege von 2007 gegenübergestellt werden [11].

Bezüglich der Einrichtung und gesetzlichen Verankerung von Gesundheitskiosken wie in INVEST Billstedt/Horn weisen Folttmann und Kießling [33] darauf hin, dass diese keine Gesundheitsversorgung im Sinne des SGB V leisten, und schlagen vor, diese an den Gesundheitsämtern anzusiedeln und damit in die Planungshoheit der Kommunen zu legen [33]. Im Rahmen der „Studie zum Versorgungsmanagement durch Patientenlotsen“ des IGES-Instituts wurden konkrete rechtliche Möglichkeiten geprüft, wie Lotsen flächendeckend in die Regelversorgung integriert werden können [12].

Das vorliegende Scoping-Review hat mehrere Limitationen. Auf inhaltlicher Ebene ist zu bemerken, dass nur Erkenntnisse aus der summativen Evaluation betrachtet wurden, die formative Evaluation allerdings wichtige Schlüsse bezüglich der Implementierungskosten und -hürden für Lotsen zulassen würde, die in Überlegungen zur Übernahme der Lotsen in die Regelversorgung eingehen könnten. Methodische Limitationen ergeben sich aus dem Fokus auf Projekte des Innovationsfonds, wodurch Projekte etwa des Bundesministeriums für Forschung, Technologie und Raumfahrt (BMFTR), des Bundesministeriums für Gesundheit (BMG) oder der Deutschen Forschungsgemeinschaft (DFG) in dem vorliegenden Review nicht berücksichtigt wurden. Es wurden ausschließlich die Ergebnis- und Evaluationsberichte für die Datenextraktion herangezogen. Zusätzliche Publikationen der Studiengruppen zur Wirksamkeit der Interventionen wurden nicht recherchiert, hätten aber gegebenenfalls vertiefte Einblicke etwa in Subgruppenanalysen ermöglicht. Es fand weiterhin keine kritische Bewertung der in den Projekten durchgeführten Evaluationen statt, sodass etwa Aussagen zur Bonferroni-Korrektur bei multiplen Outcomes nicht möglich sind. Außerdem wurde die Extraktion der Projektcharakteristika und Ergebnisse auf mehrere Personen innerhalb der Projektgruppe verteilt, sodass eine Prüfung stets nur stichprobenartig stattfand.

## Fazit

Zusammenfassend lässt sich sagen, dass sich vom Innovationsfonds geförderte Lotsenprojekte hinsichtlich der Aufgaben der Lotsen, ihrer Einsatzgebiete, der gemessenen Endpunkte und der ermittelten Effekte zu stark unterscheiden, um eine generelle Aussage bzgl. der Wirksamkeit von Lotsen im deutschen Gesundheitssystem zu treffen. Für Personen mit komplexen Versorgungsbedarfen, etwa aufgrund von Multimorbiditäten, zeigen sich durchaus positive Effekte auf PROMs, PREMs und Prozessindikatoren, für psychisch kranke Personen auch klinische Effekte. Auffällig sind außerdem unerwartete negative Effekte vor allem hinsichtlich der Kosteneffektivität der Lotsen. Eine Vereinheitlichung des Reportings zu Lotsenprojekten, die präzisere Beschreibung der Lotsentätigkeiten sowie eine verstärkte Nutzung von Wirkmodellen könnten helfen, die Wirksamkeit von Lotsen in Zukunft besser zu verstehen. Die Ergebnisse des vorliegenden Reviews können genutzt werden, um in Zukunft Lotseninterventionen zu entwickeln, die zielgruppenspezifische Wirkungen entfalten können und somit auch das Potenzial haben, in die Regelversorgung übernommen zu werden. Dazu wäre eine ähnlich holistische Betrachtung der Ergebnisse aller Projekte zu einer Neuen Versorgungsform wie die hier vorliegende durch den Innovationsausschuss notwendig.

## Supplementary Information

ESM1: Zusatzmaterial 1

## Data Availability

Alle im Rahmen der vorliegenden Studie analysierten Daten sind in dem vorliegenden Artikel enthalten.
