# Supplementary material for: Characteristics and effectiveness of healthcare navigators in innovation fund projects: a scoping review
Source: Bundesgesundheitsblatt Gesundheitsforschung Gesundheitsschutz. 2025 Dec 19;69(2):158–71. [Article in German] doi: 10.1007/s00103-025-04177-4 (PMC12852287; doi:10.1007/s00103-025-04177-4)
Supplement: Supplementary file 1 — ESM1: Zusatzmaterial 1 [file 103_2025_4177_MOESM1_ESM.pdf]

Onlinematerial

Tabelle A1: Eingeschlossene Projekte nach Bundesländern der Projektleitung

Bundesland	Projekte
Baden-Württemberg	GeriNoVe
Bayern	TIGER
Berlin	BGM-innovativ, TRANSLATE-NAMSE, Cardiolotse
Brandenburg	IGiB-StimMT
Hamburg	KID-PROTEKT, NWGA, INVEST- Billstedt/Horn, StroCare, RECOVER, TransFIT
Hessen	PREMA
Mecklenburg-Vorpommern	HerzEffektMV
Niedersachsen	MSTVK, NierenTx 360°
Nordrhein-Westfalen	SCHMERZ-NETZ, Familien-SCOUT, NPPV, STROKE OWL, RubiN, MUK
Rheinland-Pfalz	MamBo, VeMaWuRLP, HandinHand, DemStepCare, OSCAR
Saarland	PIKKO, StärkeR
Sachsen-Anhalt	IKK IVP
Schleswig-Holstein	CED Bio-Assist

Tabelle A2: Berufsgruppenzugehörigkeit der Lotsen innerhalb der Projekte

Berufsgruppenzugehörigkeit	Projekte
examiniertes Pflegepersonal	DemStepCare, IGiB-StimMT, IKK IVP, NierenTx 360°, TIGER, STROKE OWL, GeriNoVe, PIKKO, INVEST- Billstedt/ Horn, StroCare, MamBo, HerzEffektMV, VeMaWuRLP, BGM-innovativ, HandinHand, OSCAR, RubiN, TransFIT, Cardiolotse
Sozialarbeiter oder sozialpädagogische Fachkraft	IKK IVP, STROKE OWL, GeriNoVe, KID-PROTEKT, NWGA, SCHMERZ-NETZ, Familien- SCOUT, RECOVER, OSCAR
Medizinische Fachangestellte	PREMA, IKK IVP, NierenTx 360°, CED Bio-Assist, StärkeR, BGM-innovativ, RubiN, Cardiolotse
Therapeuten (z. Bsp. Physio- und Logotherapie)	MUK, STROKE OWL, MSTVK, BGM-innovativ, RubiN
Ärzte	TRANSLATE-NAMSE, MSTVK
Sozialversicherungsfachangestellte / Krankenkassenfachwirt	BGM-innovativ
Gerontologen	NWGA
Unklar	NPPV

Tabelle A3: Rechtsgrundlage für den Einsatz der Lots:innen innerhalb der Projekte

Rechtsgrundlage	Projekte
Selektivverträge nach §140a SGB V (+ ggf. Behandlungsvertrag nach §630a BGB)	DemStepCare, IGiB-StimMT, PREMA, TRANSLATE-NAMSE, IKK gesund plus, NierenTx 360°, TIGER, STROKE OWL, CED Bio-Assist, NWGA, PIKKO, StärkeR, Familien-Scout, INVEST-Billstedt/ Horn, StroCare, RECOVER, HerzEffektMV, VeMaWuRLP, OSCAR, RubiN, Cardiolotse, TransFit
Selektivvertrag § 33 in Verbindung mit § 43 Absatz 1 SGB V	MUK
Behandlungsvertrag nach §630a BGB	KID-PROTEKT
Modellvorhaben nach § 63, 64 Abs. 1 SGB V	MamBo, HandinHand
§ 43 SGB V (ergänzende Leistungen zur Rehabilitation), §1 Satz 4 SGB V (Solidarität und Eigenverantwortung)	BGM-innovativ
Nicht näher definierte Kooperationsverträge zwischen Kooperationspartnern	SCHMERZ-NETZ
Unklar	GeriNoVe, MSTVK, NPPV

Tabelle A4: Outcomes

**Effektkategorie**	**Outcome**	**Signifikant positiver Effekt**	**Positive Tendenz**	**Signifikant negativer Effekt**	**Negative Tendenz**	**Kein Effekt**	**Unklare Wirksamkeit**
Klinische Outcomes							
	Gesundheitssituation in Bezug auf geriatrische Aspekte					Rubin	
	(Ko-)Morbidität					NWGA	
	Mortalität					Cardiolotse, StroCare, STROKE OWL	NWGA, IKK IVP
	Schweregrad Erkrankung					RECOVER	
Physische Outcomes	Amputationsrate					VeMaWuRLP	
	Chronische Wunden					TIGER	
	Extraintestinale Manifestation			CED Bio-Assist			
	Funktionelle Remission					RECOVER	
	Funktioneller Status/ Ausmaß der Behinderung nach	StroCare				STROKE OWL	

	Schlaganfall						
	Gewichtsstabilisierung	NierenTx360°					
	Globale Funktionsfähigkeit					RECOVER	
	Heilungsrate		VeMaRLP (PE)				
	Kardiovaskuläre Fitness	NierenTx360°					
	Krankheitsaktivität	CED Bio-Assist				StärkeR (PE1)	
	MACE I (Herzinfarkt, Schlaganfall, kardiovaskulärer Tod)	HerzEffektMV (PE2)					
	MACE II (Herzinfarkt, Schlaganfall, kardiovaskulärer Tod, kardiovaskuläre Dekompensation)	HerzEffektMV (PE3)					
	Mangelernährung	TIGER	Rubin				
	Müdigkeit/ Fatigue					StärkeR	
	Physische Funktionsfähigkeit					StärkeR, MSTVK (PE1)	
	Pragmatische Kommunikationsfähigkeit					MUK (PE)	

	Schlaganfallrezidiv					StroCare, STROKE OWL	
	Schwerwiegende unterwünschte Ereignisse					RECOVER	
	Steroidfreie Remission			CED Bio-Assist			
	Stürze					StärkeR	Rubin
	Symptomatische Remission					RECOVER	
	Transplantationsassoziierten Komplikationen		NierenTx360° (PE)				
Psychische Outcomes	Angst/ Angstzustände	SCHMERZ-NETZ	Familien-SCOUT (PE1), PREMA			VeMaWuRLP, HerzEffektMV, StroCare, PIKKO	
	Angstbezogenes Vermeidungsverhalten					PREMA	
	Anzahl Panikattacken		PREMA				
	Depressivität	HerzEffektMV, StroCare, SCHMERZ-NETZ	Familien-SCOUT (PE2), PREMA	PIKKO		VeMaWuRLP, StärkeR, IGiB-StimMT	
	Kognitive Einschätzung		Rubin				

	Psycho-soziales Funktionsniveau		RECOVER (PE)				
	Schweregrad der Panikattacken		PREMA				
Vitalwerte	Auftreten des C-reaktiven Proteins					StärkeR	
	Blutdruck					STROKE OWL	
	Blutsenkungsgeschwindigkeit					StärkeR	
	Blutzuckerspiegel					STROKE OWL	
	Cholesterinspiegel					STROKE OWL	
PROMs							
Lebensqualität und Gesundheitszustand	Gesundheitsbezogene Lebensqualität der Patienten (allgemein)	OSCAR (nach 6 Monaten), VeMaWuRLP, HerzEffektMV, RECOVER, NPPV, SCHMREZ-NETZ	OSCAR (nach 12 Monaten) (PE), CED Bio-Assist, PREMA		Rubin, GeriNoVe (PE2)	DemStepCare, IGiB-StimMT, TransFIT, Cardiolotse, Familien-SCOUT, StärkeR (PE2), PIKKO (PE), NWGA, STROKE OWL, MUK, INVEST Billstedt/Horn (PE3)	

	Krankheitsspezifische Lebensqualität	HerzEffektMV	CED Bio-Assist (PE)			VeMaWuRLP, NPPV	
	Physische (körperliche) Lebensqualität	NierenTx360°	IKK IVP			StroCare (PE)	
	Psychische (mentale) Lebensqualität					StroCare, NierenTx 360°, IKK IVP	
	(Selbst eingeschätzter) Gesundheitszustand	HandinHand				MSTVK (PE3), INVEST Billstedt/Horn	
Kompetenzen	Gesundheitskompetenz	IGiB-StimMT	OSCAR			PIKKO, INVEST Billstedt/Horn	TRANSLATE-NAMSE
	Nutzungskompetenz der Kommunikationshilfen	MUK					
	Selbstmanagementkompetenz	GeriNoVe, CED Bio-Assist				TransFIT	
Alltägliche Tätigkeiten	Alltagsbewältigung und Selbstständigkeit		Rubin (PE)				
	Aktivität des alltäglichen Lebens					STROKE OWL	
	Bewältigung täglich notwendiger Verrichtungen im				Rubin		

	Haushalt						
	Dauer der Morgensteifigkeit					StärkeR	
	Körperliche Aktivität	NierenTx360°				STROKE OWL	
	Mobilität/ Mobilitätseinschränkungen	Rubin	GeriNoVe			TIGER	
	Raucherstatus					STROKE OWL	
	(Soziale) Teilhabe/ Teilhabeeinschränkung		CED Bio-Assist			MUK, MSTVK	
	Subjektive Arbeitsfähigkeit					MSTVK	
	Subjektive Prognose der Erwerbstätigkeit						MSTVK
Schmerzen	(Chronische) Schmerzen	BGM-innovativ, SCHMERZ-NETZ (PE1)					
	(Schmerzbezogene) Selbstwirksamkeit	SCHMERZ-NETZ				BGM-innovativ, PIKKO, KID-PROTEKT	
	Schmerzintensität	SCHMERZ-NETZ (PE2)				MSTVK	
Psychische Belastung	Kohärenz		OSCAR				

	Psychische Gesundheit		MSTVK (PE2), PREMA (PE)			KID-PROTEKT	
Adhärenz	Einstellung zur Medikation					HerzEffektMV	
	Medikations- und Therapieadhärenz	SCHMERZ-NETZ, NierenTx360°	PREMA			HerzEffektMV, CED Bio-Assist, MamBo (PE3)	
	Informations- und Wissenstransfer						TRANSLATE-NAMSE
	Patientenaktivierung					HerzEffektMV, INVEST Billstedt/Horn (PE2)	
	Patientensicherheit						HandInHand
PREMS							
Präferenzen	Beteiligungspräferenz					OSCAR	
	Informationspräferenz	OSCAR					
Zufriedenheit und Wohlbefinden	Allgemeine Patientenzufriedenheit	HandInHand	MSTVK			StärkeR, KID-PROTEKT	
	Nutzungszufriedenheit und -akzeptanz	GeriNoVe (PE1)					

	Wohlbefinden		HerzEffektMV, MamBo (PE4)				
	Zufriedenheit mit der eigenen Gesundheit	HandInHand					
	Zufriedenheit mit der Versorgung/ Therapie/ Hilfsmittel	KID-PROTEKT, CED Bio-Assist, SCHMERZ-NETZ, MUK, IGiB-StimMT, INVEST Billstedt/Horn (PE1)					PIKKO
Interaktion zwischen Ärzten und Patienten	Arzt-Patienten-Kommunikation					OSCAR	
	Entscheidungskonflikte		OSCAR				
Prozessindikatoren							
Ambulante Versorgung	Ambulante Rehabilitationsaufenthalte					StärkeR	
	Ambulante psychotherapeutische Interventionen					RECOVER	
	Ärztliche Hausbesuche					HandInHand	
	Hausarztkonsultationen					HandInHand (PE2)	

	Inanspruchnahme/ Nutzungsgrad von ambulanten Versorgungsleistungen	OSCAR	DemStepCare		NWGA	StärkeR	
	Inanspruchnahme der Online-Selbsthilfe/ Web-basierter Therapie		NPPV			RECOVER	
	Inanspruchnahme von Notfallambulanzen				IGiB-StimMt		
	Psychotherapeutische Gruppeninterventionen/ Gruppenangebote					RECOVER, NPPV	
	Psychotherapeutische Kurzzeitinterventionen					RECOVER	
	Spezifische psychiatrische Interventionen					RECOVER	
Stationäre Versorgung	Ambulant-sensitive Krankenhausfälle					INVEST Billstedt/Horn	IGiB-StimMT
	Akutstationäre Leistungsinanspruchnahme		StroCare				

	Dauer der psychiatrische Krankenhausaufenthalte					RECOVER	
	Inanspruchnahme von stationären Versorgungsleistungen			OSCAR	TransFIT (PE), NPPV	StärkeR	
	Inanspruchnahme von teilstationären Versorgungsleistungen		OSCAR		NPPV		
	Krankenhauseinweisungen/ Hospitalisierungsrate	Cardiolotse (PE), VeMaWuRLP, NierenTx 360°, IGiB-StimMT (PE)				DemStepCare, HandInHand (PE1), CED Bio-Assist, STROKE OWL (PE), TIGER (PE), IKK IVP (PE3), INVEST Billstedt/Horn	NWGA
	Krankenhausverweildauer/ stationäre Behandlungstage	MamBo, Cardiolotse, OSCAR, VeMaWuRLP				DemStepCare (PE), HerzEffektMV (PE1)	GeriNoVe
	Psychiatrische Krankenhausaufenthalte					RECOVER	
	Stationäre Rehabilitationsaufenthalte					StärkeR	

	Vermeidbare Krankenhausaufenthalte	MamBo					
Versorgungskontinuität und -stabilität	Behandlungsabbruch					RECOVER	
	Behandlungskontinuität	NPPV				RECOVER	
	Dauer bis zur Heilmittelversorgung					IKK IVP (PE1)	
	Diagnosedauer						TRANSLATE-NAMSE
	Nachsorge-Adhärenz	NierenTx360°					
	Vermeidung von Versorgungsbrüchen und -abbrüchen		TransFIT				
	Versorgungsstabilität					DemStepCare	
	Verzögerung der Behandlung					RECOVER	
	Wartezeit zwischen akuter und Reha-Behandlung						StroCare
	Weiterleitungsquote	KID-PROTEKT (PE2)					
Leitliniengerechtigkeit der Versorgung	Angemessenheit der medikamentösen Behandlung	DemStepCare					

Inanspruchnahme weiterer Versorgungsleistungen	Glukokortikosteroidgebrauch					CED Bio-Assist	
	Leitliniengerechte Behandlung					RECOVER	
	Leitliniengerechte Demenzdiagnostik und -therapie					DemStepCare	
	Leitliniengerechte Medikation					IKK IVP (PE2)	
	Sekundärprophylaxe (leitliniengerechte Pharmakotherapie)	STROKE OWL					
	Anzahl verordneter Hilfsmittel					StärkeR	
	Arzneimittelverschreibungen	OSCAR					
	Erbrachte Leistungen						GeriNoVe
	Inanspruchnahme ärztlicher Leistungen					SCHMERZ-NETZ	
	Inanspruchnahmequote		KID-PROTEKT (PE1)				
	Inanspruchnahme von Gesundheitsleistungen (GKV)						SCHMERZ-NETZ
	Inanspruchnahme von Heilmitteln					StärkeR	

	Inanspruchnahme von Pflegeleistungen und häusliche Betreuungsleistungen		StroCare			NWGA, StärkeR	
	Medikamentenkonsum					SCHMERZ-NETZ	
Qualität der Versorgung	Anzahl gesicherter Diagnosen	TRANSLATE-NAMSE (PE)					
	Arzneimitteltherapiesicherheit					NWGA	
	Behandlungsqualität in Krisensituationen		NPPV				
	Bewertung der medizinischen Versorgung		PREMA				
	Versorgungsqualität	PREMA	MamBo (PE1)			NPPV	
	Zugang zur Gesundheitsversorgung	INVEST Billstedt/ Horn					
Gesundheitsökonomische Faktoren							
Kosteneffektivität und Gesamtkosten	Gesamtkosten	Cardiolotse	IGiB-StimMT	HandInHand	PREMA	StärkeR, MamBo	GeriNoVe
	GKV-Leistungsausgaben	OSCAR	HerzEffektMV	NPPV (PE)	TransFIT, CED Bio-Assist	INVEST Billstedt/Horn	

	Kosteneffektivität	RECOVER, KID-PROTEKT, Cardiolotse	HerzEffektMV, IGiB-StimMT		DemStepCare, Rubin, Familien-SCOUT	NWGA	VeMaWuRLP, TIGER, PREMA
	Versorgungs-, Krankheits- und Behandlungskosten	RECOVER		STROKE OWL	Familien-SCOUT	StroCare, TIGER	
Spezifische Leistungsausgaben	Ambulante Kosten		IGiB-StimMT		PREMA, MamBo	CED Bio-Assist	PIKKO
	Arzneimittelkosten		IGiB-StimMT		MamBo	CED Bio-Assist	PIKKO
	Kosten für Heil- und Hilfsmittel					CED Bio-Assist	
	Pflegekosten			STROKE OWL			
	Psychotherapiekosten						PIKKO
	Stationäre Kosten		IGiB-StimMT, MamBo	CED Bio-Assist	PREMA	NierenTx 360°	
Arbeitsunfähigkeit	Arbeitsfähigkeit					BGM-innovativ (PE1)	
	AU-Tage					RECOVER, NPPV, SCHMERZ-NETZ, BGM-innovativ (PE2)	VeMaWuRLP, PIKKO

	AU-Kosten					CED Bio-Assist	
	Ausgaben pro Monat					SCHMERZ-NETZ	
	Finanzielle Belastung durch die Erkrankung					SCHMERZ-NETZ	
	Indirekte Kosten						PREMA
Patientenbezogene Versorgungssituation							
Angehörige und Familie	Belastung pflegender Angehöriger			Rubin		DemStepCare	
	Elterliche Belastung (mind. 1 Elternteil pro Familie)	Familien-SCOUT					
	Familiäre Belastung					TransFIT	
	Lebensqualität pflegender Angehöriger					DemStepCare	
	Peer-Unterstützung		RECOVER				
	Resilienz pflegender Angehöriger					DemStepCare	
	Soziale Unterstützung bei Krankheit	MamBo (PE2)				HerzEffektMV	
	Stress pflegender Angehöriger					DemStepCare	

Pflege- und Hilfebedürftigkeit	Hilfebedürftigkeit		GeriNoVe				
	Pflegebedürftigkeit					NWGA, STROKE OWL	IKK IVP (PE4)
	Progression der Pflegebedürftigkeit					NWGA (PE)	

Anmerkungen: PE – primärer Endpunkt der (patientenbezogenen) Effektivitätsstudie; Projekt, die mehr als einen primären Endpunkt genannt haben, wurden entsprechen durchnummeriert

Tabelle A5: Outcomes und Effekte nach Zielgruppen

	**Outcomes gesamt**	**positiv (Anteil)**	**kein Effekt (Anteil)**	**negativ (Anteil)**	**unklar (Anteil)**
Psychische und Verhaltensstörungen (5 Projekte)	55	19 (34,6 %)	26 (47,3 %)	8 (14,6 %)	2 (3,6 %)
Krankheiten des Kreislaufsystems (5 Projekte)	42	15 (35,7 %)	23 (54,8 %)	2 (4,8 %)	2 (4,8 %)
Multimorbidität (3 Projekte)	42	15 (35,7 %)	18 (42,9 %)	9 (21,4 %)	0 (0,0)
Neubildungen (2 Projekte)	28	13 (46,4 %)	6 (21,4 %)	4 (14,3 %)	5 (17,9 %)
alle Patienten mit Nierentransplant	10	8 (80,0 %)	2 (20,0 %)	0 (0,0 %)	0 (0,0 %)
Symptome und abnorme klinische und Laborbefunde, die anderenorts nicht klassifiziert sind	13	7 (53,9 %)	5 (38,5 %)	0 (0,0 %)	1 (7,7 %)
Krankheiten des Verdauungssystems	19	5 (26,3 %)	5 (26,3 %)	6 (31,6 %)	3 (15,8 %)
alte Menschen mit akut sozial-pflegerischem Versorgungsbedarf	5	4 (80,0 %)	0 (0,0 %)	1 (20,0 %)	0 (0,0 %)
Familien mit psychosozialen Belastungen durch Erkrankungen der Kinder	7	4 (57,1 %)	3 (42,9 %)	0 (0,0 %)	0 (0,0 %)
Krankheiten des Nervensystems	10	4 (40,0 %)	6 (60,0 %)	0 (0,0 %)	0 (0,0 %)
schlecht heilende (chronischen) Wunden	10	4 (40,0 %)	4 (40,0 %)	0 (0,0 %)	2 (20,0 %)
Majoramputation der unteren Gliedmaßen	8	2 (25,0 %)	5 (62,5 %)	0 (0,0 %)	1 (12,5 %)
Krankheiten des Muskel-Skelett-Systems und des Bindegewebes (2 Projekte)	20	1 (5,0 %)	19 (95,0 %)	0 (0,0 %)	0 (0,0 %)
Patient*innen, die mindestens das Alter von 75 Jahren erreicht haben	6	1 (16,7 %)	4 (66,7 %)	0 (0,0 %)	1 (16,7 %)
Seltene Erkrankungen	4	1 (25,0 %)	0 (0,0 %)	0 (0,0 %)	3 (75,0 %)
Personen <70 Jahre mit Risiko für Hilfs- und Pflegebedürftigkeit	10	0 (0,0 %)	6 (60,0 %)	2 (20,0 %)	2 (20,0 %)

Tabelle A6: Ergebnisse der Projekte mit Transferempfehlung durch den Innovationsausschuss

Projekt	Positive Effekte auf...	Keine Effekte auf...	Negative Effekte auf...	Unklare Effekte auf...
Familien-SCOUT	• Angst/ Angstzustände<br>• Depressivität<br>• Elterliche Belastung (mind. 1 Elternteil pro Familie)	• Gesundheitsbezogene Lebensqualität der Patienten (allgemein)	• Kosteneffektivität<br>• Versorgungs-, Krankheits- und Behandlungskosten	
IGiB-StimMT	• Gesundheitskompetenz<br>• Zufriedenheit mit der Versorgung/ Therapie/ Hilfsmittel<br>• Krankenhauseinweisungen/ Hospitalisierungsrate<br>• Gesamtkosten<br>• Kosteneffektivität<br>• Ambulante Kosten<br>• Arzneimittelkosten<br>• Stationäre Kosten	• Depressivität<br>• Gesundheitsbezogene Lebensqualität der Patienten (allgemein)	• Inanspruchnahme von Notfallambulanzen	• Ambulant-sensitive Krankenhausfälle
INVEST-Billstedt/Horn	• Zufriedenheit mit der Versorgung/ Therapie/ Hilfsmittel<br>• Zugang zur Gesundheitsversorgung	• Gesundheitsbezogene Lebensqualität der Patienten (allgemein)<br>• (Selbst eingeschätzter) Gesundheitszustand<br>• Gesundheitskompetenz<br>• Patientenaktivierung<br>• Ambulant-sensitive Krankenhausfälle<br>• Krankenhauseinweisungen/		

		Hospitalisierungsrate<br>• GKV-Leistungsausgaben		
SCHMERZ-NETZ	• Angst/ Angstzustände<br>• Depressivität<br>• (Chronische) Schmerzen<br>• (Schmerzbezogene) Selbstwirksamkeit<br>• Schmerzintensität<br>• Medikations- und Therapieadhärenz<br>• Zufriedenheit mit der Versorgung/ Therapie/ Hilfsmittel	• Inanspruchnahme ärztlicher Leistungen<br>• Medikamentenkonsum<br>• AU-Tage<br>• Ausgaben pro Monat<br>• finanzielle Belastung durch die Erkrankung		• Inanspruchnahme von Gesundheitsleistungen (GKV)
TRANSLATE-NAMSE	• Anzahl gesicherter Diagnosen			• Gesundheitskompetenz<br>• Informations- und Wissenstransfer<br>• Diagnosedauer
